# Prevalence of prognostic factors for cancer of the uterine cervix after radical hysterectomy

**DOI:** 10.1590/S1516-31802009000300007

**Published:** 2009-10-06

**Authors:** Marília Buenos Aires Cabral Tavares, Rodrigo Beserra Sousa, Thiago Oliveira e Silva, Larissa Almeida Moreira, Loyana Teresa Teófilo Lima Silva, Carolina Buenos Aires Cabral Tavares, Sabas Carlos Vieira

**Affiliations:** 1 MD. General Surgery Resident, Hospital Universitário Walter Cantídio (HUWC), Universidade Federal do Ceará, Fortaleza, Ceará, Brazil.; 2 Medical student, School of Medicine, Universidade Federal do Piauí (UFPI), Teresina, Piauí, Brazil.; 3 MD. Trained at Universidade Federal do Piauí (UFPI), Teresina, Piauí, Brazil.; 4 MD. Gynecologist and Obstetrician, Maternidade-Escola Assis Chateaubriand (MEAC), Universidade Federal do Ceará (UFC), Fortaleza, Ceará, Brazil.; 5 MD, PhD. Oncological Surgeon and Professor, Universidade Federal do Piauí (UFPI), Teresina, Piauí, Brazil.

**Keywords:** Survival analysis, Cervix uteri, Uterine neoplasms, Hysterectomy, Gynecologic surgical procedures, Análise de sobrevida, Colo do útero, Neoplasias uterinas, Histerectomia, Procedimentos cirúrgicos em ginecologia.

## Abstract

**CONTEXT AND OBJECTIVE:**

Cancer of the uterine cervix is still very common in Brazil. It is important to evaluate factors that influence its prognosis. The aim here was to analyze the prevalence of prognostic anatomoclinical factors among patients with carcinoma of the uterine cervix undergoing radical hysterectomy.

**DESIGN AND SETTING:**

Cross-sectional study on 301 patients with invasive carcinoma of the uterine cervix who underwent Level III Piver-Rutledge hysterectomy surgery at São Marcos Hospital.

**METHODS:**

The following variables were analyzed: age, histological type, degree of differentiation, invasion of lymphatic, vascular and perineural space, lymph node metastasis, distance to nearest margin, tumor invasion depth, vaginal cuff size, largest diameter of the tumor, presence of necrosis and surgical margin involvement. Descriptive statistics, multiple regression analysis, Kaplan-Meier survival curves and the log-rank test were performed. A significance level of 5% was used.

**RESULTS:**

The mean age was 48.27 years. The following were not important for the prognosis, in relation to survival analysis: degree of differentiation and tumor invasion depth; presence of lymphatic, blood and perineural invasions; distance to nearest margin; and vaginal cuff size. Tumor size (P < 0.036), presence of lymph node metastasis (P < 0.0004), necrosis (P < 0.05) and surgical margin involvement (P < 0.0015) presented impacts on survival. The overall survival with 98 months of follow-up was 88.35%.

**CONCLUSION:**

The most prevalent prognostic factors were the presence of lymph node metastasis, tumor size and surgical margin involvement.

## RESUMO

**CONTEXTO E OBJETIVO:**

O câncer do colo do útero é ainda muito frequente no Brasil, sendo importante avaliar os fatores que influenciam no seu prognóstico. O objetivo é analisar a prevalência dos fatores prognósticos anatomoclínicos em pacientes com carcinoma do colo uterino submetidas a histerectomia radical.

**TIPO DE ESTUDO E LOCAL:**

Estudo retrospectivo de 301 pacientes com carcinoma invasivo de colo uterino submetidas a histerectomia radical nível III de Piver-Rutledge no Hospital São Marcos.

**MÉTODOS:**

Analisaram-se as variáveis: idade, tipo histológico, grau de diferenciação, invasão do espaço linfático, vascular e perineural, metástase linfonodal, distância da margem mais próxima, profundidade de invasão do tumor, tamanho do manguito vaginal, maior diâmetro do tumor, presença de necrose, comprometimento de margens cirúrgicas. Realizou-se estatística descritiva, análise de regressão múltipla, curva de sobrevida pelo método de Kaplan-Meier e teste log rank. Adotou-se índice de significância de 5%.

**RESULTADOS:**

A idade média foi de 48,27 anos. A análise da sobrevida em relação ao grau de diferenciação e profundidade de invasão do tumor, à presença de invasões linfática, sanguínea e perineural, à distância da margem mais próxima e ao tamanho do manguito vaginal não foram importantes no prognóstico. O tamanho do tumor (P < 0,036), presença de metástase linfonodal (P < 0,0004), necrose (P < 0,05) e comprometimento de margens cirúrgicas (P < 0,0015) apresentaram impacto na sobrevida. A sobrevida global com um acompanhamento de 98 meses foi de 88,35%.

**CONCLUSÃO:**

Os fatores prognósticos mais prevalentes foram a presença de metástase linfonodal, o tamanho do tumor, a presença de necrose e o comprometimento das margens cirúrgicas.

## INTRODUCTION

Cervical cancer is a serious public health issue in Latin America. In Brazil, it is the third most common malignant type of cancer among women, only surpassed by skin cancer (non-melanoma) and breast cancer. Moreover, it is the greatest cause of death among the female population of reproductive age. This type of cancer represents 10-15% of all malignant tumors in women.^1^^,^^2^ In the State of Piauí, Brazil, cervical cancer is ranked second to skin cancer (non-melanoma), among the most frequent types of cancer affecting women.^3^^,^^4^ In 2004, this state and its capital presented an increasing trend of mortality due to cervical cancer among the age group of 20 years and over, following regional behavior in this respect. Brazil showed stable rate trends.^3^^,^^4^

The treatment of choice for invasive cervical cancer in the initial stages, i.e. stages Ib1 and Ib2 of the International Federation of Gynecology and Obstetrics (FIGO), is radiotherapy or radical hysterectomy with pelvic bilateral lymphadenectomy. Surgery is preferable among young patients because of the possibilities of ovary preservation, hormonal function maintenance and better sexual performance.^5^ Anatomopathological evaluation of the surgical specimen after radical hysterectomy makes it possible to determine prognostic factors that define whether complementary therapy is needed.^6^^,^^7^^,^^8^^,^^9^^,^^10^^,^^11^^,^^12^^,^^13^^,^^14^^,^^15^^,^^16^

Patients with large tumors in stages Ib and IIa have a greater tendency towards lymph node metastasis and shorter survival, regardless of whether treated by radical hysterectomy or by radiotherapy.^8^^,^^9^ There is greater incidence of lymph node metastasis in the presence of deep stromal invasion, along with smaller rates of two-year disease-free survival (58% versus 8%), than among patients without stromal invasions and with negative pelvic lymph nodes.^6^^,^^17^ The presence of positive lymph nodes *per se* decreases disease-free survival considerably, falling from 80.4% when there is no lymph node involvement to 60.4% when there are three compromised lymph nodes and to 45.9% among patients with four or more compromised lymph nodes.^6^

However, the prognosis is also affected by the histological type, degree of cell differentiation and other patient-related factors. Adenocarcinoma is considered to have the worst prognosis. Tumor size, vascular and lymph node involvement, depth of invasion, parametrial invasion, surgical margin involvement, age group, histological type and degree of tumor differentiation are factors that can also influence the prognosis.^6^^,^^7^

## OBJECTIVES

The objective of the present study was to analyze the prevalence of prognostic anatomoclinical factors among patients with uterine carcinoma undergoing radical hysterectomy.

## METHODS

In this investigation, a convenience sample was used, consisting of 301 patients with cervical cancer in FIGO stages I and II who underwent radical hysterectomy at São Marcos Hospital, carried out by the same surgeon (Vieira SC), between July 1999 and July 2006. All of the patients underwent the same surgical procedure: Level III Piver-Rutledge hysterectomy, with bilateral pelvic lymphadenectomy. The inclusion criteria were: age less than 45 years, regular menstrual cycles, absence of hypoestrogenism symptoms (heat waves or vaginal dryness) and macroscopically normal ovaries as observed during the surgery. Our institution’s Ethics Committee approved the investigation.

The surgical procedure consisted of laparotomy with a medial or upper transverse pubic incision. Level III Piver-Rutledge hysterectomy was performed, with linking of the origin of the uterine artery and parametrium, followed by colpectomy. Bilateral pelvic lymphadenectomy was performed with the following boundaries: crossing of the urethra with the iliac vessels (upper limit); psoas muscles (sides); urethra (medial limit); obturator nerve (back); and origin of the circumflex artery in the iliac artery (lower limit). The pelvic peritoneum was left open, and all the patients received pelvic drainage using a vacuum suction drain with the external counter-aperture at the abdominal cavity. Prophylaxis with antibiotics (cefazoline; 1g every three hours) was administered, from the time when anesthesia was induced. All the patients received subcutaneous heparin as prophylaxis for deep vein thrombosis. Postoperative radiotherapy was indicated in cases of presence of compromised lymph nodes, compromised vaginal margins or margins of less than 0.5 cm and parametrial involvement.

The ovaries of 59 patients were preserved. Verbal consent concerning ovary preservation was sought. The preserved ovaries were held at the parietal-colic gutter, homolateral to the level of bifurcation of the aorta, and were clipped with metal clamps, while always observing whether the vascular ovarian pedicle was correctly positioned, in order to avoid torsion and/or traction.

The patients were followed up every four months, over the first two years, and then every six months, from the second to the fifth year. The routine included speculum examination, digital rectal examination and onco-cytological tests on material collected from the vaginal dome. In the presence of specific symptoms, tests were ordered in accordance with the patients’ clinical symptoms.

The following variables were analyzed: age group, histological type, lymph node space invasion, vaginal cuff size, largest diameter of the tumor, presence of necrosis and surgical margin involvement (when parietal involvement was found).

The Winstat^®^ software was used to perform descriptive statistical analysis, multiple regression analysis to determine the importance of prognostic factors, Kaplan-Meier survival curve analysis and the log-rank test. A significance level of 5% was used.

## RESULTS

The patients’ mean age was 48.27 years, with a range from 25 to 83 years. The mean length of follow-up was 24.57 months, with a range from 0.3 to 98 months. The mean depth of invasion was 1.54 cm. The mean vaginal cuff size was 1.28 cm. The mean distance to the closest margin was 0.83 cm. Fifty-nine patients (19.6%) of the study group had at least one preserved ovary. The most frequent histological type was squamous cell carcinoma. Undifferentiated tumors were more frequent. In most cases, there was no blood, lymphatic or neural invasion from the tumor. Furthermore, in most cases, there was no lymph node metastasis or surgical margin involvement. Necrosis was usually present in cases with neoplasia (Table 1).

**Graph 1.**
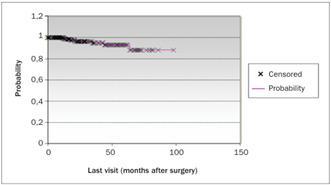
Overall survival of all the 301 patients in the study with a follow up of 98 months, using the Kaplan-Meier method.

**Graph 2.**
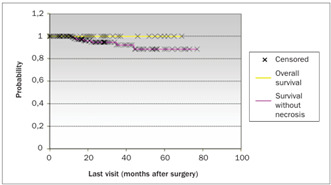
Overall survival of all 310 patients in the study compared with survival in the group without tumor necrosis, using the log-rank test and Kaplan-Meier method.

**Graph 3.**
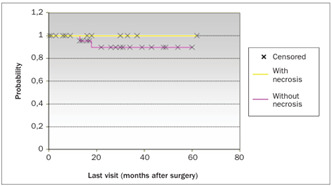
Survival of the groups with and without tumor necrosis, using the log-rank test and Kaplan-Meier method.

**Graph 4.**
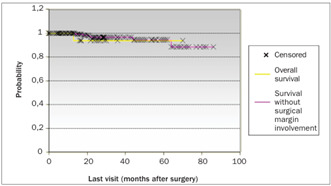
Overall survival of all 310 patients in the study compared with survival in the group without surgical margin involvement, using the log rank test and Kaplan-Meier method.

**Graph 5.**
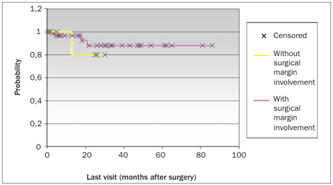
Survival of the groups with and without surgical margin involvement, using the log-rank test and Kaplan-Meier method.

The patients’ ages, degree of differentiation, tumor invasion depth, presence of blood, lymphatic and neural invasion from the tumor, distance to the nearest margin and vaginal cuff size were not important for the prognosis. The largest diameter of the tumor (P < 0.04), presence of lymph node metastasis (P < 0.01), necrosis (P < 0.05) and surgical margin involvement (P < 0.01) presented impacts on the patients’ survival rate (Table 2). Graph 1 shows the overall survival (involving all the patients in the study) with a follow-up of 98 months was 88.35%. The log rank test was used to compare the overall survival with the survival of the patients with and without tumor necrosis and with and without surgical margin involvement. Graph 2 compares the overall survival over 77 months (88.58%) with the survival of the group without tumor necrosis, which was 100% over a follow-up of 69 months. Graph 3 compares the survival of this group without tumor necrosis with the survival of the group with tumor necrosis, which was 89% over a follow-up of 60 months. Graph 4 compares the overall survival (involving all the patients in the study) with the survival in the group without surgical margin involvement, which was 93.75% over a follow-up of 60 months. Because of the number of cases, it was also possible to compare the survival between groups with and without surgical margin involvement, over a follow-up of 30 months, as shown in Graph 5. Over this period, the survival in the group with surgical margin involvement was 80%, while it was 88% in the group without surgical margin involvement. Of these, 92 (30.57%) only had radiotherapy, two (0.66%) only had chemotherapy and 20 (6.64%) had both radiotherapy and chemotherapy.

**Table 1.** Characteristics of neoplasms from 301 Brazilian patients with cancer of the uterine cervix

Characteristics		%
Histological type	Spindle cell carcinoma	70
	Adenocarcinoma	21
	Squamous adenocarcinoma	8
	Unknown*	1
Difference degree	Well differentiated	18
	Moderately differentiated	30
	Poorly differentiated	42
	Unknown*	10
Blood invasion	Yes	18
	No	82
Lymphatic invasion	Yes	29
	No	71
Neural invasion	Yes	27
	No	73
Lymph node metastasis	Yes	14
	No	86
Compromised surgical margins	Yes	8
	No	92
Necrosis	Yes	78
	No	22

*Data not located.

**Table 2.** Variables with statistical significance for survival among 301 patients with cancer of the uterine cervix

Variable	Significance (P)
Largest tumor diameter	P < 0.04
Lymph node metastasis	P < 0.01
Presence of necrosis	P < 0.05
Surgical margin involvement	P < 0.01

## DISCUSSION

It has already been established that many factors influence the prognosis for cervical cancer patients undergoing radical hysterectomy. Among these factors are the degree of pelvic lymph node involvement with tumor cells, the size of the tumor, vascular involvement, depth of invasion, parametrial invasion, surgical margin involvement, age, histological type and degree of tumor differentiation.^6^^,^^7^^,^^8^^,^^9^^,^^17^

In this study, we found that the factors influencing the survival of the patients who underwent radical hysterectomy were the presence of lymph node metastasis, tumor size and surgical margin involvement. Furthermore, we found that a variable rarely described in the medical literature was a factor influencing the patients’ survival, namely the degree of necrosis on the tumor presented in the histological examination.

The presence of lymph node metastasis is the single most important prognostic factor for cervical carcinoma.^6^^,^^8^ It is known that lymph drainage from the uterine cervix tends to go to compromised pelvic lymph nodes. Therefore, their compromised nature increases the possibility of distant pelvic metastatic relapses, while not diminishing the chances of dissemination and pelvic relapse.^6^^,^^8^ We also found that the size of the tumor was another important influence on patients’ prognoses. This gives rise to the consensus that the larger the tumor is, the worse the prognosis.^6^^,^^8^^,^^9^ Tumors smaller than 2.0 cm form a low-risk group, with a disease-free five-year survival rate of 95%. On the other hand, tumors larger than 2.0 cm form a high-risk group and, in tumors larger than 4.0 cm, the survival rate falls to 52%.^8^^,^^9^^,^^12^^,^^15^^,^^16^ Surgical margin involvement was another factor that influenced the patients’ prognoses. In previous studies, this has been described as another very important prognostic factor. This may be because surgical margin involvement means that some tumor remains were probably not removed, which creates more possibilities of larger relapses and metastasis.^6^^,^^7^^,^^8^^,^^9^^,^^15^

An important finding to which our attention was drawn during the investigation was the presence of necrosis on the tumor. This was observed under the microscope, from sections stained with hematoxylin-eosin. This factor had a negative influence on the patients’ survival. This finding has rarely been described in the literature, although data confirming that the presence of tumor necrosis is associated with lymph node metastasis do exist.^18^ Nevertheless, the real influence of this factor on cervical cancer patients’ prognoses needs to be studied in more detail.

It is relevant to note that some factors that are often described as important prognostic factors were not statistically significant in the present investigation. Among these were the patients’ ages, degree of tumor differentiation, histological type of the tumor, tumor size, blood, neural and lymphatic invasion, distance to closest margin and vaginal cuff size. This perhaps happened because of the short follow-up. However, it needs to be highlighted that the importance of lymphatic and vascular invasion is still controversial: there is evidence to corroborate the results obtained in this study, as well as the opposite.^6^^,^^8^^,^^9^^,^^10^^,^^11^^,^^13^^,^^14^

## CONCLUSION

In this study, the prognostic factors that were most prevalent among patients with cervical cancer who underwent radical hysterectomy were the presence of necrosis in lymph node metastases, tumor size, surgical margin involvement and the presence of necrosis of the tumor. The first three factors have already been consolidated in the literature, but the last one is a factor that has only recently been considered important for the survival of patients undergoing this surgery.
